# Steering Photoelectrons Excited in Carbon Dots into Platinum Cluster Catalyst for Solar‐Driven Hydrogen Production

**DOI:** 10.1002/advs.201700273

**Published:** 2017-09-21

**Authors:** Xiaoyong Xu, Wenshuai Tang, Yiting Zhou, Zhijia Bao, Yuanchang Su, Jingguo Hu, Haibo Zeng

**Affiliations:** ^1^ College of Physics Science and Technology Yangzhou University Yangzhou 225002 China; ^2^ Institute of Optoelectronics and Nanomaterials College of Materials Science and Engineering Nanjing University of Science and Technology Nanjing 210094 China

**Keywords:** carbon dots, charge transfer, hydrogen evolution, photocatalysis

## Abstract

In composite photosynthetic systems, one most primary promise is to pursue the effect coupling among light harvesting, charge transfer, and catalytic kinetics. Herein, this study designs the reduced carbon dots (r‐CDs) as both photon harvesters and photoelectron donors in combination with the platinum (Pt) clusters and fabricated the function‐integrated r‐CD/Pt photocatalyst through a photochemical route to control the anchoring of Pt clusters on r‐CDs' surface for solar‐driven hydrogen (H_2_) generation. In the obtained r‐CD/Pt composite, the r‐CDs absorb solar photons and transform them into energetic electrons, which transfer to the Pt clusters with favorable charge separation for H_2_ evolution reaction (HER). As a result, the efficient coupling of respective natures from r‐CDs in photon harvesting and Pt in proton reduction is achieved through well‐steered photoelectron transfer in the r‐CD/Pt system to cultivate a remarkable and stable photocatalytic H_2_ evolution activity with an average rate of 681 µmol g^−1^ h^−1^. This work integrates two functional components into an effective HER photocatalyst and gains deep insights into the regulation of the function coupling in composite photosynthetic systems.

## 1 Introduction

Hydrogen (H_2_) energy is clean and considered as one of the most promising alternatives for fossil‐based energy in the future.^1^, ^2^ Photocatalytic water splitting into H_2_ and oxygen (O_2_) using solar energy has attracted considerable attention as a green, low‐cost, and sustainable approach for large‐scale H_2_ production.^3^, ^4^, ^5^, ^6^ Photocatalytic H_2_ evolution reaction (HER) proceeds a consistent route that protons in solution are reduced by photoexcited electrons to hydrogen atoms chemisorbed on catalyst surface followed by their desorption into hydrogen gas.^7^, ^8^ Thus, the injection of thermodynamic photoelectrons and the Gibbs free energy of atomic H adsorption (Δ*G*_H_) are two critical factors in determining the photocatalytic HER activities of catalysts.^9^ Platinum (Pt) yields the most desirable Δ*G*_H_ of near‐zero value (−0.09 eV),^10^, ^11^ while its photochemical inertness toward sunlight restricts the potential for direct photocatalysis. In recent years, the heterogeneous systems equipped with efficient coupling of solar photon harvesting and photoinduced charge transfer with catalysis kinetics have shown great success for solar photocatalytic applications.^12^, ^13^, ^14^, ^15^ In particular, the low‐cost and nontoxic carbon dots (CDs) have recently received significant interest as an effective function unit in designing composite photocatalysts.^16^, ^17^, ^18^, ^19^, ^20^ For example, Kang and co‐workers reported the loading of CDs on traditional semiconductor photocatalysts and demonstrated the promising results for the photocatalytic and photo‐electrochemical water splitting.^21^, ^22^, ^23^ Metal nanoparticles like gold (Au) and Pt have also been developed to integrate with CDs to form composite photocatalysts with high efficiency and high selectivity for the oxidation of cyclohexane^24^ and the reduction of carbon dioxide.^25^ Recently, Reisner and co‐workers reported a smart photocatalytic HER system using CDs as a photosensitizer in combination with a molecular Ni catalyst.^26^ Along with these pioneering results, CDs were demonstrated to possess the excellent photon harvesting and photoinduced electron transfer properties. However, to the best of our knowledge, CDs have been underexplored to combine with Pt catalyst for photocatalytic H_2_ production; moreover, there have been a few efforts devoted to control CDs on light absorption character and electron transfer mode (acceptor or donor) for specific photocatalysis.

**Figure 1:**
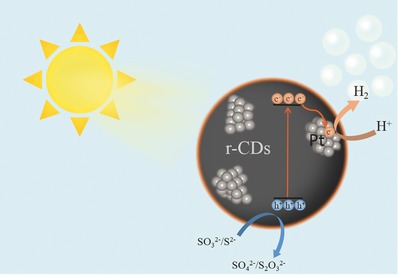
Schematic diagram of solar H_2_ evolution over r‐CD/Pt composite catalyst. Photoexcited electrons in r‐CDs transfer to Pt clusters in contact and drive aqueous proton reduction; photogenerated holes in r‐CDs were consumed by oxidation reaction with Na_2_SO_3_ and Na_2_S.

Herein, in order to combine the attractive properties of both CDs and Pt, we synthesized a composite photocatalyst via loading of Pt clusters onto surface‐reduced CDs (r‐CDs), and then tested its photocatalytic activity for solar H_2_ evolution. The r‐CD/Pt composite shows promise as an effective and stable platform for converting aqueous protons into H_2_ under solar irradiation. Moreover, the efficient coupling between excellent properties of r‐CDs in solar photon harvesting and Pt in proton reduction was demonstrated to cultivate an artificial HER activity in the r‐CD/Pt composite based on well‐steered photoinduced electron transfer from r‐CDs to Pt clusters (**Figure** **1**).

## 2 Results and Discussion

**Scheme 1:**
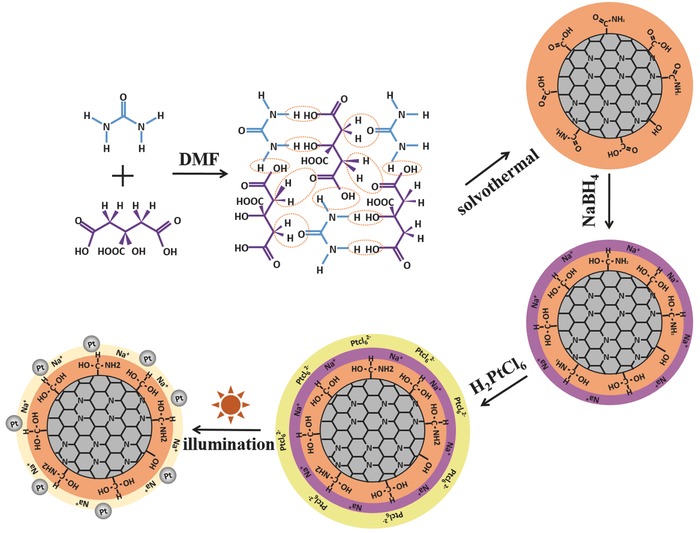
Schematic representation of the synthetic procedure for r‐CD/Pt composite.

The r‐CD/Pt composite was fabricated through the synthesis route shown in **Scheme** **1** (see the “Experimental Section” for full details). In brief, 2–5 nm large CDs with rich surface groups were first prepared by the dehydrolysis reaction between urea and citric acid in dimethylformamide (DMF) solvent^27^ and used as precursor materials. Then, sodium borohydride (NaBH_4_) as a reducing agent was introduced into the CD solution to reduce the surface carbonyl/carboxyl species and to modify surface charge by adsorbing Na^+^ cations. The surface components of CDs before and after treated with NaBH_4_ were analyzed in Figure S1 (Supporting Information) in terms of the Fourier transform infrared (FT‐IR) and the X‐ray photoelectron spectroscopies (XPS). From FT‐IR spectra in Figure S1a (Supporting Information), the as‐synthesized CDs are found to be hybridized with abundant oxygenous groups, such as hydroxyl, carbonyl, and carboxyl groups, on their surfaces. Because oxygen atoms are more electronegative than carbon atoms,^28^ the negatively charged surface can form and then inhibit the electron output as well as the electrostatic adsorption of Pt anionic species.^29^ Hence, the NaBH_4_ was used as a reducing agent to reduce carbonyl and carboxyl groups, inducing an increased amount of hydroxyl groups on the surface. As expected, in the FT‐IR spectrum of r‐CDs, the absorption band of C=O stretching vibration at 1710 cm^−1^ almost quenches, whereas the absorption band at 3000–3600 cm^−1^ of O—H stretching vibration increases when compared to that of CDs.^30^, ^31^ Moreover, the XPS spectra of C 1s region (Figure S1c,d, Supporting Information) also display that the C=O peak at 288.9 eV obviously declines and the —OH peak at 286.0 eV raises after the reduction treatment, while the conjugated sp^2^ carbon peak at 284.6 eV is almost unchanged.^32^ In addition, the full‐range XPS spectra (Figure S1b, Supporting Information) reveal the presence of C, N, O, and Na in the r‐CDs, whereas no Na signal is detected in the pristine CDs. These analyses for chemical composition suggest the surface reduction and charge modification making r‐CDs release electron‐donating ability, which is crucial for anchoring Pt clusters and steering photoelectron transfer toward Pt clusters. Next, the purified r‐CDs were added to H_2_PtCl_6_ aqueous solution to allow the electrostatic adsorption of Pt anionic species on r‐CDs as supports. The Pt‐ion‐adsorbing r‐CDs were extracted by centrifugation and re‐dissolved in deionized water to remove the excess Pt species. Finally, the above solution of r‐CDs absorbed with Pt ions was irradiated under sunlight for 60 min, and the solution color varied from dark brown to light yellow, indicating the transformation of Pt ions to atomic clusters. In the contrast experiments for photochemical Pt deposition (Figure S2, Supporting Information), the unreduced CDs were found to fail to anchor Pt species, whereas the r‐CDs operated the continuous Pt deposition from the precursor H_2_PtCl_6_ solution under light irradiation, further confirming that only the r‐CDs can allow for Pt species anchoring and steer photoelectron donating for Pt nucleation growth. In previous reported works,^26^, ^33^ the CDs conjugated with carbonyl (C=O) and carboxyl (COOH) groups were also found to be unfavorable for the photochemical deposition of metallic Pt, probably because more negative surface restricts the electron overflowing. In addition, note that the photochemical Pt deposition in the H_2_PtCl_6_ solution was difficult to be controlled, so we implemented a two‐step route, that is, the separated chemical adsorption of Pt anionic species and photoreduction of metallic Pt, for the control of ultrafine Pt cluster anchoring on r‐CDs' surface to avoid the Pt growth or aggregation.

**Figure 2:**
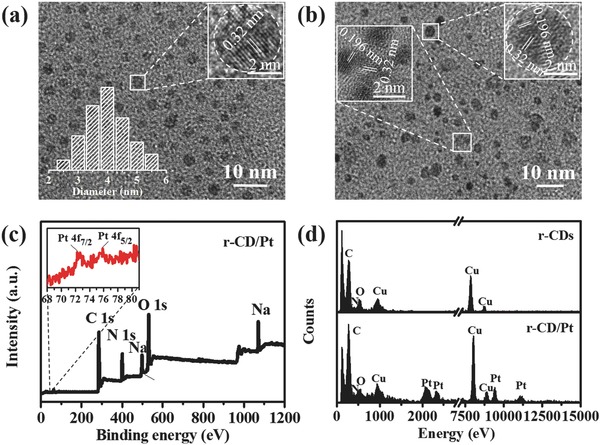
TEM and HRTEM (inset) images of a) r‐CDs and b) r‐CD/Pt. Lower left inset in panel (a) shows the size distribution of r‐CDs. c) Full‐survey XPS spectrum with magnification for Pt 4f region (inset) and d) EDX spectra of r‐CD/Pt as well as r‐CDs as a reference.

As shown in **Figure** **2**a, the transmission electron microscopy (TEM) image of as‐synthesized r‐CDs clearly shows the monodispersed distribution of 2–5 nm large particles with an average size of around 4.0 nm (lower left inset). The high‐resolution TEM (HRTEM) image (upper right inset) shows the circular shape of r‐CDs, with visible crystalline lattice fringes. The clear crystal domain from typical individual reveals the lattice fringes of graphite (002) plane with an interlayer spacing of 0.32 nm.^34^ The ultrafine size favors the shortening of the route of photogenerated charge carrier migrating toward the surface for consecutive reactions. The monodispersed distribution renders a smart platform for the subsequent loading of Pt clusters. Figure 2b shows the TEM image of r‐CD/Pt composite, depicting the major production with the size range of 4–8 nm and no aggregation, even if the uniformity of particle size decreases due to the loading of Pt. The HRTEM image of r‐CD/Pt (inset) describes two clear lattice spacings of 0.32 and 0.196 nm, which can be indexed to the graphite (002) plane and Pt (200) plane,^35^ indicating the successful loading of ultrafine Pt clusters with the size less than 2 nm onto r‐CDs' surface. Furthermore, the intimately intersecting between two groups of different lattice fringes in the binding region supports the nucleation of Pt clusters on r‐CDs, which ensures the strong connection and the convenient electron transfer across r‐CD/Pt interface. The XPS spectrum of r‐CD/Pt composite in Figure 2c shows two extremely weak signals of Pt 4f_7/2_ and 4f_5/2_ at 72.7 and 76.1 eV at the magnified Pt 4f region,^36^ indicating the especially low Pt loading. The energy‐dispersive X‐ray (EDX) spectrum (Figure 2d) acquired from individual r‐CD/Pt particle along with the HRTEM characterization present the clear emergence of Pt component compared to that from pure r‐CDs, which further determine the formation of r‐CD/Pt composite, where the estimated Pt loading as low as ≈0.38 wt% is amenable to economization of Pt usage (Table S1, Supporting Information).

**Figure 3:**
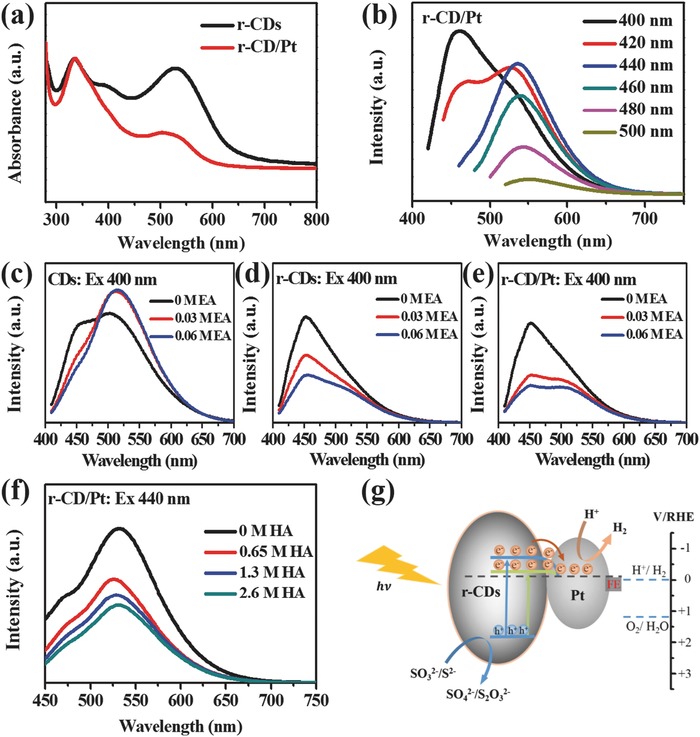
a) UV–vis absorption spectra of r‐CDs and r‐CD/Pt. b) PL spectra of r‐CD/Pt at different excitation wavelengths. c–e) PL spectra (400 nm excitation) of CDs, r‐CDs, and r‐CD/Pt in aqueous solutions with the varying concentrations of quenchers (EA, K_2_S_2_O_8_) and f) PL spectra (440 nm excitation) of r‐CD/Pt in aqueous solutions with the varying concentrations of quenchers (HA, Na_2_S/Na_2_SO_3_). g) Schematic of the proposed photocatalytic mechanism of proton reduction over r‐CD/Pt catalyst.

The photoinduced charge transition and transfer properties were investigated based on the ultraviolet–visible (UV–vis) absorption and photoluminescence (PL) spectroscopic analyses. By comparing the UV–vis absorption spectra in **Figure** **3**a, it can be seen that r‐CD/Pt composite inherits the optical absorption feature of r‐CDs that the broad optical absorption covers the UV and visible regions while the pure Pt particles almost do not absorb any light. The absorption peak at ≈335 nm represents a typical π–π* transition in r‐CDs, similar to that of previously reported CDs,^37^ while the absorption shoulder in the visible region is attributed to the surface trap states. The unreduced CDs exhibit the multipeak absorption in the visible region (Figure S3a, Supporting Information), corresponding to the existence of various surface states, and show typical excitation‐wavelength‐dependent PL behavior (Figure S3c, Supporting Information).^38^ Because of the surface reconfiguration, the r‐CDs show the less excitation‐dependent PL emission peak position at around 550 nm in addition to the π–π* recombination emission at 465 nm (Figure S3d, Supporting Information). Moreover, the r‐CD/Pt composite exhibits a similar PL behavior to r‐CDs in Figure 3b, indicating that there is a unique transition model with the specific energy, in addition to π–π* transition from sp^2^ domains, dominating the optical absorption and exciton behaviors in the visible region. Therefore, it can be believed that the unit of r‐CDs involved in r‐CD/Pt composite can serve as both solar photon harvester and photoinduced charge generator to compensate the drawback of Pt catalyst in response to solar light. However, the strong PL emission occurring in CDs is adverse to photocatalysis because it represents the photogenerated charge recombination. Fortunately, the intimate contact at the interface can make the laden Pt clusters act as the electron reservoirs to allow the fast separation of photogenerated charge. The photoinduced electron transfer properties were probed through the influences of adding an electron acceptor (EA, K_2_S_2_O_8_) on the PL spectra in aqueous solution. Because of electron trapping at carbonyl groups on surface, the pristine CDs restrict the electron overflowing toward solution; thus, the corresponding PL intensity does not drop in the presence of K_2_S_2_O_8_, but appears the slight increase at 500 nm owing, likely, to the consolidated carbonyl surface (Figure 3c). In contrast, the r‐CDs shown in Figure 3d exhibit the distinct PL quenching behavior, indicating that the reduced surface indeed can release the electron‐donating capacity to allow the reaction with K_2_S_2_O_8_ in solution. Furthermore, the more effective PL quenching effect occurs in the r‐CD/Pt (Figure 3e), which demonstrates that the facile electron transfer from r‐CDs to Pt provides more probabilities for electrons to react with K_2_S_2_O_8_ in solution before being recombined with holes. Alternatively, the adding of hole acceptors (HA, Na_2_S, and Na_2_SO_3_) can also effectively retard the electron–hole recombination, resulting in the PL quenching behavior in the r‐CD/Pt solution (Figure 3f). Hence, it can be concluded that the r‐CD/Pt composite steers the favorable photoelectron transfer from r‐CDs to Pt clusters for the photogenerated charge separation and the subsequent redox reactions.

For the photocatalytic water splitting, besides the high photogenerated electron supply, the appropriate band structure with matching band edges that straddle the redox potential of water‐splitting reaction is also critical important.^39^, ^40^ Herein, we plotted the curve of (*αhν*)^2^ against *hν* to determine the band‐gap energy (*E*_g_) of r‐CD/Pt to be about 2.80 eV (Figure S4, Supporting Information), where α, *h*, and ν are the absorption coefficient, Planck constant, and light frequency, respectively. Moreover, we adopted the linear potential scan method to determine the energy levels of conduction band (CB) and valence band (VB) of r‐CDs. Applying potentials above the CB to form an accumulation layer, or below the VB to form inversion layers, can result in abrupt emergence of cathodic and anodic currents, respectively.^16^ The cathodic and anodic scan profiles (Figure S5, Supporting Information), respectively, estimate the CB and VB potentials of ≈−1.31 and 1.25 eV versus Ag/AgCl electrode, which were then converted to relative values of −0.70 and 1.86 eV to reversible hydrogen electrode (RHE) using a Nernst conversion equation $\left(E_{\mathrm{RHE}}\;\;=\;\;E_{\mathrm{Ag}/\mathrm{AgCl}}\;\;+\;\;E_{\mathrm{Ag/AgCl}}^{0}\;\;+\;\;0.059\;\;\mathrm{pH}\right)$,^41^ where $E_{\mathrm{Ag/AgCl}}^{0}$ is the standard potential of Ag/AgCl reference electrode (0.1976 V vs RHE at 25 °C), *E*_RHE_ and *E*_Ag/AgCl_ are the converted potential versus RHE and the measured potential versus Ag/AgCl. The bandgap energy estimated from the potential scans was 2.56 eV, which is close to that obtained from the optical absorption spectrum. According to the converted curve of (*αhν*)^2^ versus *hν* in Figure S4 (Supporting Information), the surface state may be positioned at an energy level of −0.08 eV versus RHE below the CB. Based on the preceding data, the energy level diagram of r‐CD/Pt is depicted in Figure 3g, with the proposed charge transfer mechanism for proton reduction to H_2_. The r‐CDs can be excited by solar light to produce electrons into the CB and surface state, and leave holes in the VB; the CB and surface state are above the reduction level for H_2_O to H_2_, endowing electrons with thermodynamic appropriateness for proton reduction; holes are consumed by sacrificial reagents while energic electrons fast transfer to the Pt clusters, where they more actively trigger proton reduction to H_2_. It is notable that the laden Pt clusters can serve as not only electron‐accepting reservoirs to promote charge separation but also highly active co‐catalysts to facilitate proton reduction reaction. Hence, the coupling among light harvesting, electron transfer, and catalytic kinetics could be achieved to develop the potential of r‐CD/Pt as a photocatalyst for solar H_2_ generation.

**Figure 4:**
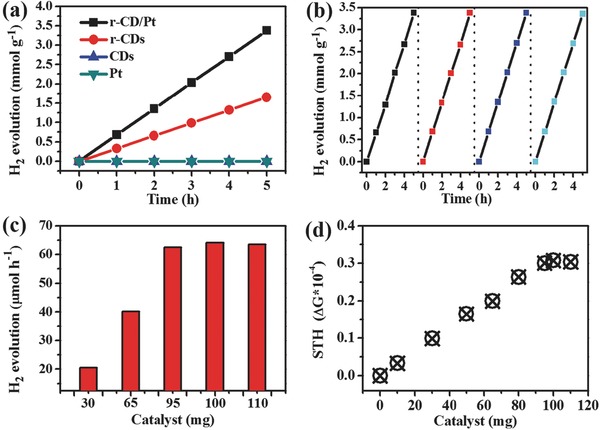
a) Time‐dependent photocatalytic H_2_ evolution for r‐CD/Pt, r‐CDs, CDs, and pure Pt particles. b) Cycling test of photocatalytic H_2_ evolution for r‐CD/Pt. c) Evolved H_2_ amounts per hour and d) STH efficiencies for r‐CD/Pt at different usage doses.

**Figure** **4**a shows the photocatalytic H_2_ evolution, respectively, using r‐CDs, r‐CD/Pt, CDs, and pure Pt particles with the same amount, 30 mg, in 100 mL of aqueous solution containing 0.5 m Na_2_SO_3_ and 0.7 m Na_2_S under the simulated sunlight irradiation from a 300 W Xe lamp with an AM 1.5G filter. The bare Pt particles do not yield any detectable H_2_ evolution due to no light absorption, and the unreduced CDs, even if have the desirable light absorption, also produce zero HER activity because of the confinement of electrons inside the carbonyl surface. Due to the electron‐donating capacity releasing, the r‐CDs render a definite HER activity, which is still limited by the rapid recombination of electron–hole pairs. As expected, the r‐CD/Pt composite with optimized electron transfer and proton reduction kinetics shows a significantly enhanced HER activity relative to bare r‐CDs. Figure 4b records the four successive cycles of photocatalytic H_2_ evolution over 30 mg of r‐CD/Pt for 20 h, where the evolved H_2_ amount almost linearly increases with irradiation time in each course without the obviously detectable activity decay. So the r‐CD/Pt composite was demonstrated to be capable of executing the stable photocatalytic H_2_ production with an average rate of 681 µmol h^−1^ g^−1^. In addition, the H_2_ output per hour can be further improved by the optimization of usage dose of r‐CD/Pt catalyst (Figure 4c). With the usage dose increasing, the H_2_ output per hour gradually increases, after that it instead decreases owing to the optical shielding effect induced by the excess suspended catalyst. The solar‐to‐hydrogen (STH) efficiencies are also shown in Figure 4d to increase with the dose of r‐CD/Pt catalyst increasing, and reach a maximum at 100 mg dose. The practical H_2_ evolution tests demonstrate that the r‐CD/Pt composite is an effective and stable photocatalyst for solar‐driven H_2_ production. Note that the superior activity results from the synergistic effect of CDs in light harvesting and Pt in proton reduction under the control of well‐steered electron transfer, because both the pristine CDs and the bare Pt are inert toward photocatalytic HER.

## 3 Conclusion

In conclusion, we reported an effective r‐CD/Pt composite photocatalyst based on the combination of two superior function components for solar H_2_ production. In the r‐CD/Pt composite, the r‐CDs are capable of harvesting solar light, generating and transferring “hot” electrons to the Pt clusters in contact; the Pt is most favorable for proton reduction reaction because of its appropriate Δ*G*_H_. Thus, an excellent and stable photocatalytic HER activity was cultivated through the efficient coupling among three attractive properties of r‐CDs in harvesting photons and donating electrons, and Pt clusters in reducing protons. This work highlights the necessity of steering photoinduced charge transfer in composite photosynthetic systems to work the synergistic action.

## 4 Experimental Section

*Syntheses of CDs, r‐CDs, and r‐CD/Pt*: The CDs hybridized with various surface groups were prepared via a solvothermal route from citric acid and urea using DMF as solvent. In a typical synthesis, 1 g of citric acid and 2 g of urea were reacted in 10 mL of DMF solvent at 160 °C for 6 h under solvothermal condition. After naturally cooled to room temperature, the obtained dark brown solution was mixed with ethyl alcohol (1:8), and then centrifuged at 12 000 rpm for 5 min. The precipitate was collected, dispersed in deionized water, and centrifuged (16 000 rpm, 5 min) twice to wash off residual salts and alkali, and then freeze‐dried to obtain the product of CDs. To prepare the surface‐reduced CDs (r‐CDs), 0.1 g of sodium borohydride (NaBH_4_) was introduced into the 10 mL of CDs aqueous solution (10 mg mL^−1^) and then stirred gently overnight at room temperature. The obtained solution was mixed with ethyl alcohol (1:8), and then centrifuged at 12 000 rpm for 5 min. The precipitate was collected, dissolved in deionized water, and centrifuged (16 000 rpm, 5 min) twice to wash off residual reducing agent, and then freeze‐dried to obtain the product of r‐CDs. Finally, a two‐step photochemical strategy was adopted to anchor the ultrafine Pt clusters on the surface of r‐CDs. As a typical procedure, 200 µL of H_2_PtCl_6_ aqueous solution (2 × 10^−3^ m) and 10 mL of r‐CDs aqueous solution (10 mg mL^−1^) was mixed and then gently stirred for 24 h at room temperature. The precipitate was collected and washed repeatedly with ethyl alcohol by centrifugation at 16 000 rpm, and then dissolved in 50 mL of deionized water. The obtained solution was irradiated under sunlight for 60 min, and the color of this solution varied from dark brown to light yellow, indicating the reduction of absorbed Pt ions to metallic Pt. The r‐CD/Pt product was repeatedly washed with ethyl alcohol and deionized water by centrifugation at 16 000 rpm for 5 min. For comparison, pure Pt particles were also synthesized via the similar process without the presence of r‐CDs as supports.

*Characterization*: TEM and HRTEM images were taken on a Tecnai F30 electron microscope equipped with the X‐ray energy dispersive spectrum. FT‐IR spectra were recorded with a Varian Cary 670 spectrometer. XPS measurements were carried out on an ESCALAB250Xi spectrometer with a monochromatic Al Kα X‐ray source operating at 150 W, and the binding energy was calibrated according to the C 1s peak at 284.6 eV. UV–vis absorption spectra were drawn by a UV–vis spectrophotometer (Varian Cary 5000) in the range from 200 to 800 nm. The PL spectra were recorded at room temperature with a luminescence spectrophotometer (Hitachi FL4600) using the Xe lamp emission with regulatory wavelength as an excitation source.

*Photocatalytic Hydrogen Evolution*: Photocatalytic hydrogen evolution experiments were carried out in a closed gas circulation and evacuation system fitted with a top window of optical flat quartz glass (Labsolar‐IV (AG), Perfectlight, Beijing). The different samples (CDs, r‐CDs, r‐CD/Pt, and pure Pt particles) with the same amount, 30 mg, were respectively employed as catalysts and dispersed in 100 mL of aqueous solution containing 0.5 m Na_2_SO_3_ and 0.7 m Na_2_S as the sacrificial agents. The suspensions were sealed in the glass vessels and purged with N_2_ for 30 min prior to photocatalytic experiments to remove dissolved oxygen. The vials were then placed in a thermoregulated rack at 26 °C with magnetic stirring and irradiated the simulated solar light from an Xe lamp with an AM 1.5G filter, and the focused light power intensity (*I*_i_) on the reactor was uniformly calibrated to 100 mW cm^−2^. The amount of evolved hydrogen was determined with an online gas chromatograph (9790 II, Fuli, Zhejiang) equipped with a thermal conductivity detector and an N_2_ gas carrier. In addition, the photocatalytic hydrogen evolution experiments of r‐CD/Pt catalyst with different usage doses (10, 30, 50, 65, 80, 95, 100, and 110 mg) were carried out to probe the maximum hydrogen yield per hour. The STH conversion efficiencies were evaluated for the different catalyst doses. The total incident power (*P*_i_) over the irradiation area (*S*_i_) of 5 cm^−2^ was *P*_i_ = *I*_i_ × *S*_i_ = 0.5 W, and the total input solar energy (*E*_i_) for 1 h of irradiation time (*t*) was *E*_i_ = *P*_i_ × *t* = 1.8 × 10^3^ J. Basing on the evolved hydrogen amount (*M*_H_), the chemical energy converted into hydrogen (*E*_H_) was calculated by using an equation *E*_H_ = *M*_H_ × ΔG, where ΔG is the certain Gibbs free energy, so that the STH could be semiquantitatively estimated according to an equation STH = (*E*_H_/*E*_i_) × 100% to obtain its change trend with the catalyst dose increasing.

## Conflict of Interest

The authors declare no conflict of interest.

## Supporting information

Supplementary
