# Supplementary material for: Steering Photoelectrons Excited in Carbon Dots into Platinum Cluster Catalyst for Solar‐Driven Hydrogen Production
Source: Adv Sci (Weinh). 2017 Sep 21;4(12):1700273. doi: 10.1002/advs.201700273 (PMC5737228; doi:10.1002/advs.201700273)
Supplement: Supplementary file 1 — Supplementary [file ADVS-4-na-s001.pdf]

Copyright WILEY-VCH Verlag GmbH & Co. KGaA, 69469 Weinheim, Germany, 2017.

ADVANCED SCIENCE
Open Access

Supporting Information

for *Adv. Sci.,* DOI: 10.1002/advs.201700273

Steering Photoelectrons Excited in Carbon Dots into Platinum Cluster Catalyst for Solar-Driven Hydrogen Production

Xiaoyong Xu, Wenshuai Tang, Yiting Zhou, Zhijia Bao, Yuanchang Su, Jingguo Hu,* and Haibo Zeng**

Steering Photoelectrons Excited in Carbon Dots into Platinum Cluster Catalyst for Solar-Driven Hydrogen Production

Xiaoyong Xu,[#] Wenshuai Tang,[#] Yiting Zhou, Zhijia Bao, Yuanchang Su, Jingguo Hu, and Haibo Zeng**

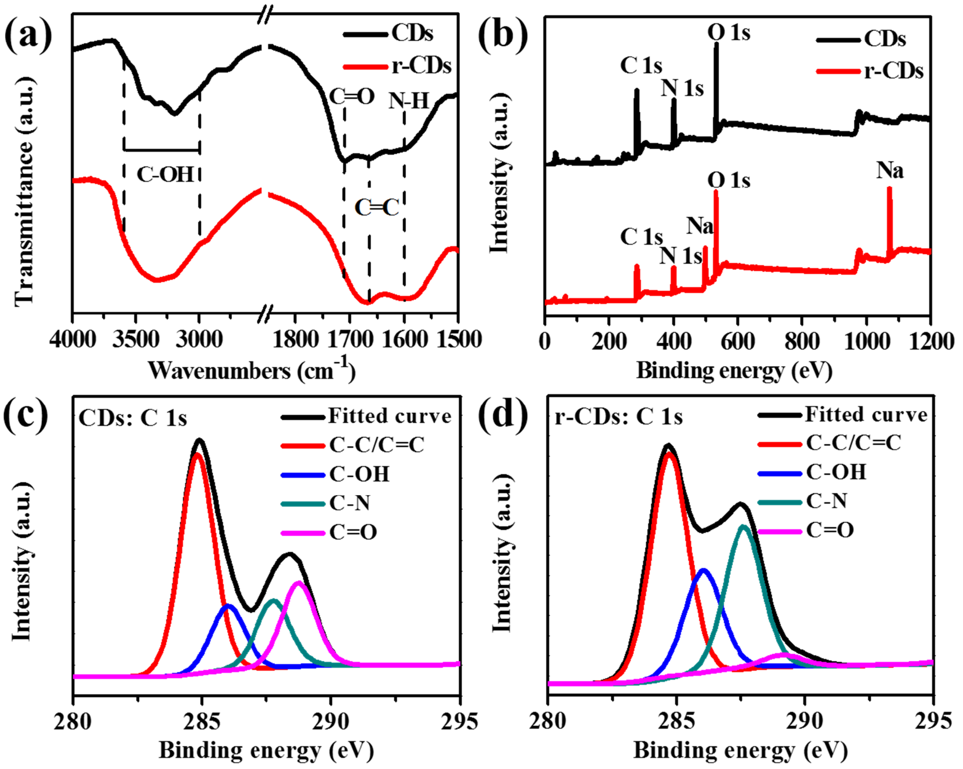

Figure S1. (a) FT-IR, (b) full-range and (c) C 1s XPS spectra of CDs and r-CDs.

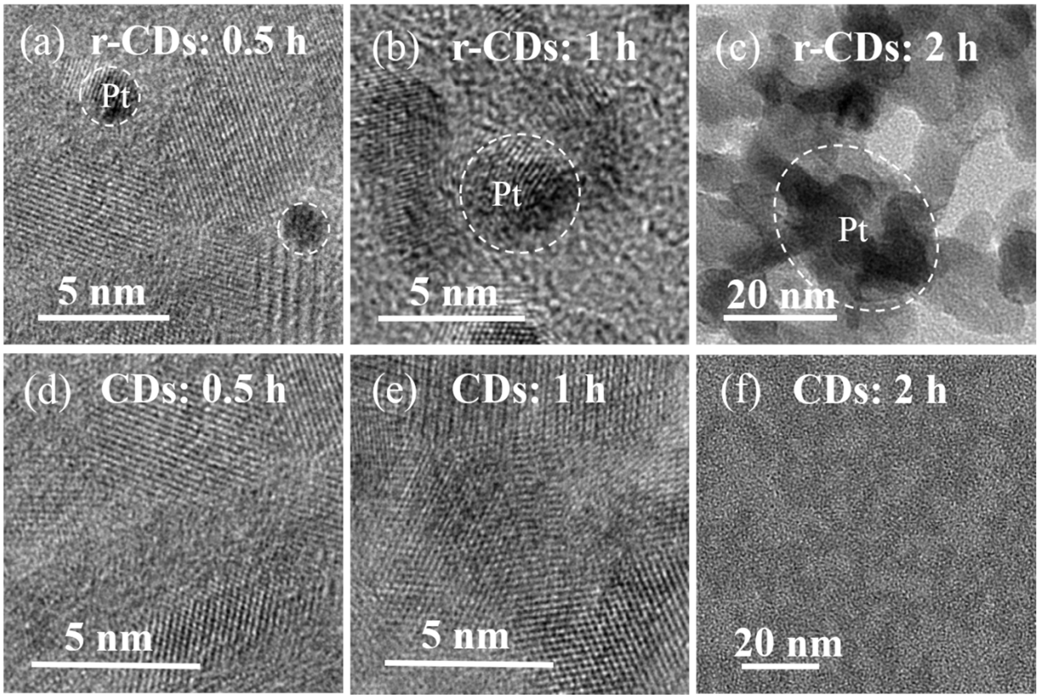

Figure S2. Photochemical Pt deposition tracking HRTEM images using (a-c) r-CDs and (d-f) CDs as supports with time under solar light irradiation, respectively.

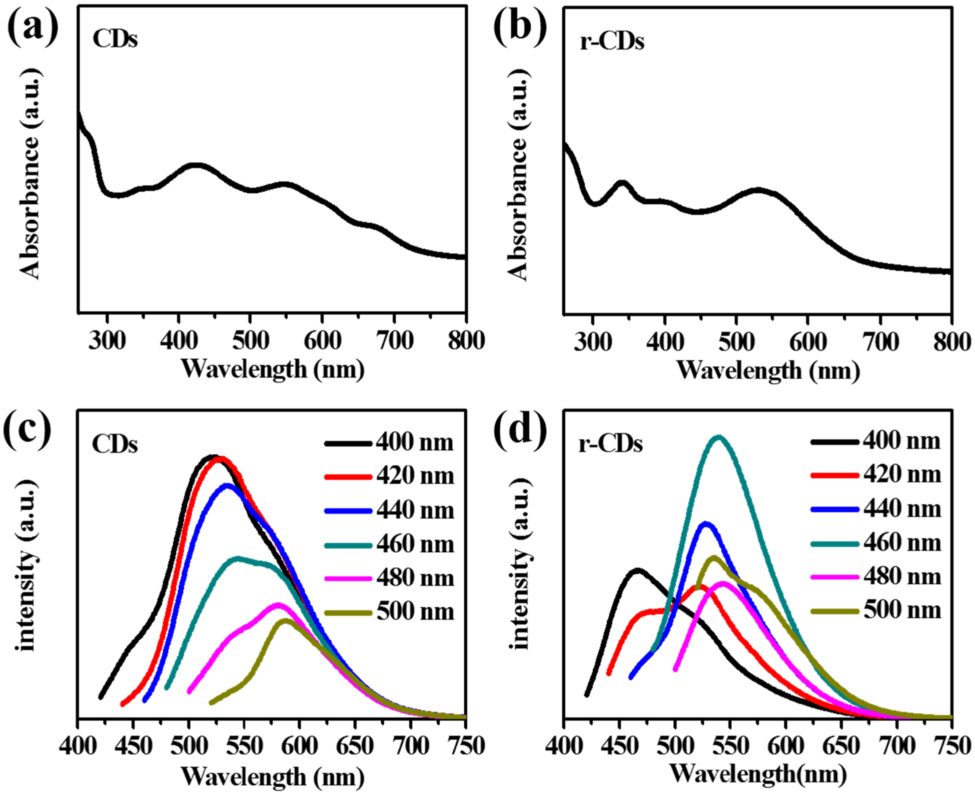

Figure S3. (a, b) UV-Vis absorption and (c, d) PL spectra of CDs and r-CDs.

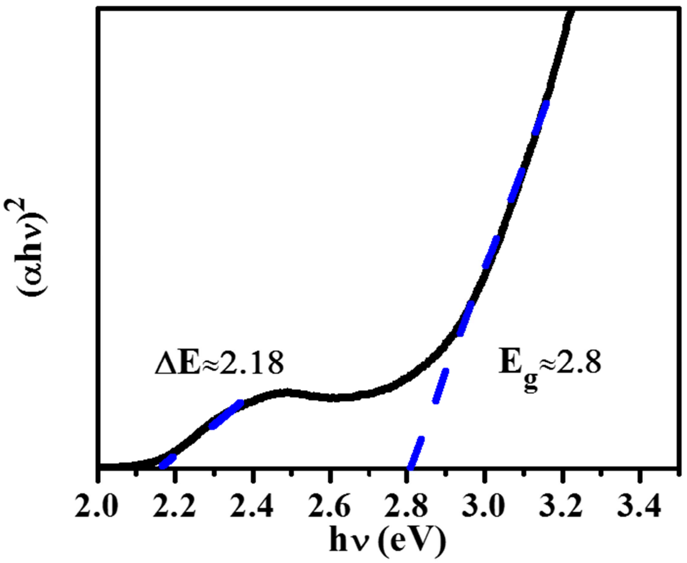

Figure S4. The calculation of band gap of r-CDs based on the $(\alpha h\nu)^2$ versus $h\nu$ curve.

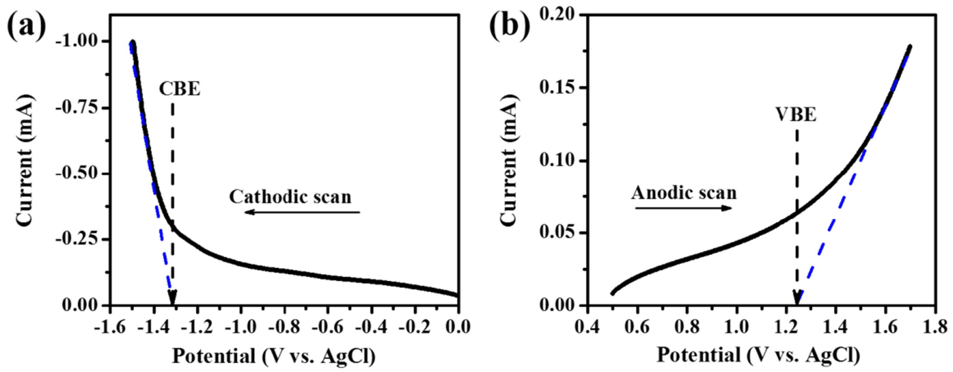

Figure S5. (a) Cathodic and (b) anodic scans for determining the energy levels of CB minimum and VB maximum, respectively.

Name	Peak BE	FWHM eV	Area(p) CPS.eV	Weight %
O 1s	531.34	3.18	199911.03	41.86
Na 1s	1071.07	3.00	167780.49	21.94
C 1s	285.35	3.32	73044.63	29.17
N 1s	399.32	3.44	22678.72	6.65
Pt 4f	72.21	1.18	212.51	0.38

Table S1. Element proportion analysis of r-CD/Pt by XPS characterization.
